# A Functional Interplay between Human Immunodeficiency Virus Type 1 Protease Residues 77 and 93 Involved in Differential Regulation of Precursor Autoprocessing and Mature Protease Activity

**DOI:** 10.1371/journal.pone.0123561

**Published:** 2015-04-20

**Authors:** Christopher J. Counts, P. Shing Ho, Maureen J. Donlin, John E. Tavis, Chaoping Chen

**Affiliations:** 1 Department of Biochemistry and Molecular Biology, Colorado State University, Fort Collins, Colorado, United States of America; 2 Department of Molecular Microbiology and Immunology, Saint Louis University School of Medicine, St. Louis, Missouri, United States of America; 3 Department of Biochemistry and Molecular Biology, Saint Louis University School of Medicine, St. Louis, Missouri, United States of America; 4 Saint Louis University Liver Center, Saint Louis University School of Medicine, St. Louis, Missouri, United States of America; Centro de Biología Molecular Severo Ochoa (CSIC-UAM), SPAIN

## Abstract

HIV-1 protease (PR) is a viral enzyme vital to the production of infectious virions. It is initially synthesized as part of the Gag-Pol polyprotein precursor in the infected cell. The free mature PR is liberated as a result of precursor autoprocessing upon virion release. We previously described a model system to examine autoprocessing in transfected mammalian cells. Here, we report that a covariance analysis of miniprecursor (p6*-PR) sequences derived from drug naïve patients identified a series of amino acid pairs that vary together across independent viral isolates. These covariance pairs were used to build the first topology map of the miniprecursor that suggests high levels of interaction between the p6* peptide and the mature PR. Additionally, several PR-PR covariance pairs are located far from each other (>12 Å Cα to Cα) relative to their positions in the mature PR structure. Biochemical characterization of one such covariance pair (77–93) revealed that each residue shows distinct preference for one of three alkyl amino acids (V, I, and L) and that a polar or charged amino acid at either of these two positions abolishes precursor autoprocessing. The most commonly observed 77V is preferred by the most commonly observed 93I, but the 77I variant is preferred by other 93 variances (L, V, or M) in supporting precursor autoprocessing. Furthermore, the 77I93V covariant enhanced precursor autoprocessing and Gag polyprotein processing but decreased the mature PR activity. Therefore, both covariance and biochemical analyses support a functional association between residues 77 and 93, which are spatially distant from each other in the mature PR structure. Our data also suggests that these covariance pairs differentially regulate precursor autoprocessing and the mature protease activity.

## Introduction

The human immunodeficiency virus 1 (HIV-1) protease (PR) is initially synthesized as part of the Gag-Pol polyprotein precursor in the infected cell. Within the Gag-Pol precursor, the PR is flanked at the N-terminus by a transframe region (TFR)—also called p6* as it overlaps with the p6 late domain of Gag—and at the C-terminus by the reverse transcriptase [1–3]. The PR domain while embedded in the Gag-Pol polyprotein has proteolysis activity that is essential and sufficient for processing of the Gag-Pol precursor to release the free, fully active mature PR. This process is generally referred to as precursor autoprocessing, although the underlying molecular mechanism remains poorly defined [2,4,5]. The 99 amino acid mature PR exists as a stable homodimer with each monomer contributing the critical aspartate residue (D25) to form the catalytic site at the dimer interface [6,7]. The mature PR recognizes and processes multiple sites in the Gag and Gag-Pol polyproteins at different rates and is essential for the production of infectious virions [8–11].

**Fig 1:**
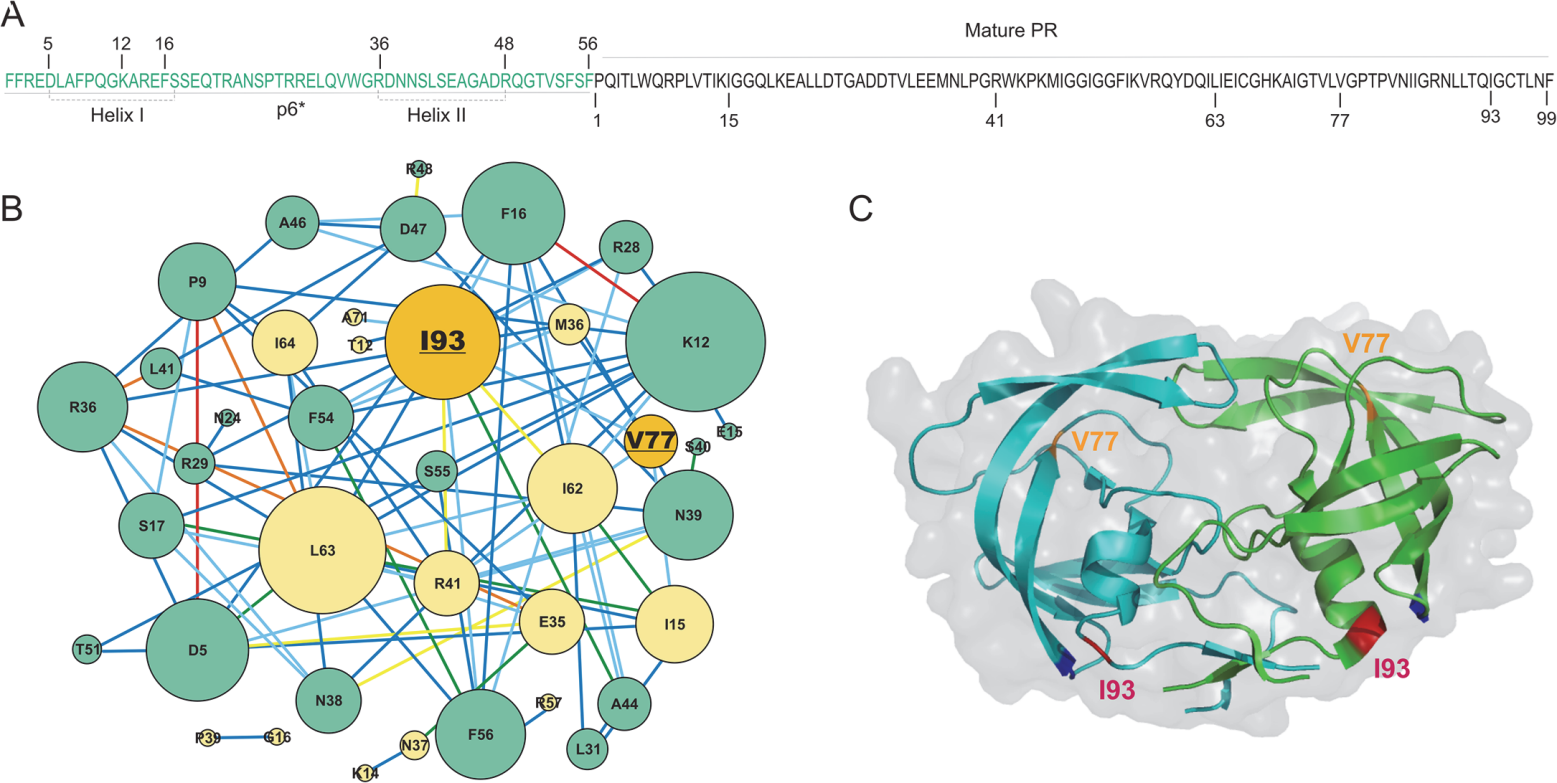
A precursor protease sequence and covariance pairs. Panel A lists the p6*-PR sequence derived from NL4-3 with the p6* sequence in green and PR sequence in black. The mature PR and p6* peptide are numbered separately. The two predicted helices in the p6* region are indicated. Panel B shows the covariance network formed by OMES analysis. Green nodes correspond to p6* residues and yellow nodes correspond to PR residues. Residue 93 and 77 are underlined and highlighted in orange. The edges are color coded per S score values as in Fig 2. Panel C demonstrates locations of V77 (orange) and I93 (red) in the context of a HIV-1 mature PR dimer structure (PDB code 3DJK).

There are at least two cleavage reactions (the N- and C-terminal cleavages, respectively) that are critical to liberate the mature PR from the precursor. The C-terminal cleavage has previously been shown to be dispensable for mature PR activity and mutations that block this cleavage result in the production of infectious viral particles [12–14]. In contrast, the N-terminal cleavage that removes the p6* peptide is indispensable for the production of mature PR and infectious virions [15–17]. Consequently, p6*-PR has been defined as a miniprecursor (Fig 1A) with the N-terminal cleavage reaction being the critical step in precursor autoprocessing [18–20].

It is intuitive to assume that the precursor and mature PR function similarly as both contain the same PR sequence. However, emerging data suggest that their catalytic mechanisms might differ. For example, the mature PR recognizes and processes all Gag and Gag-Pol cleavage sites, but the p6*-PR precursor is capable of processing fewer [5,16]. More importantly, the p6*-PR precursor is significantly less sensitive than the mature PR to inhibition by the currently available PR inhibitors [18,20–23]. Since these PR inhibitors are designed to primarily target the catalytic site of the mature PR, this difference in sensitivity to PR inhibitors suggests that the active sites and/or catalytic mechanism of these two forms of PR might be different. We previously reported that changing PR residue H69 to negatively charged amino acids (E or D) abolished precursor autoprocessing while having minimal effect on the mature PR activity [24]. Taken together, these results suggest a discernable difference between these two forms of PR and possible conformational changes that accompany precursor autoprocessing.

It is technically challenging to study whether and how the wild type precursor is structurally different from the mature PR due to its autoprocessing property. Additionally, the p6* peptide by itself appears to be intrinsically disordered, which increases the difficulty of isolating a structurally homogenous precursor. Recently, it has become apparent that this structural flexibility contributes to allosteric regulation by permitting an ensemble of conformations [25]. Therefore, it is theoretically possible that p6*-PR might exhibit more than one conformation as determined by interaction with different viral/host factors. At the moment, all the structural information is obtained from recombinant mature PRs that are expressed in *E*. *coli* and purified under denaturing conditions followed by *in vitro* refolding [15,26–28]. However, it remains unclear whether this *in vitro* folding procedure favors the formation of mature PR-like structure over other ensembles, especially in the presence of a protease inhibitor (PI). In this regard, a puzzling example is that a multi-drug-resistant mature protease carrying 20 mutations made by this approach still binds to darunavir (DRV), the most potent PI available [29], with low nM affinity, whereas in the contexts of a model precursor or the entire virus this protease demonstrates resistance to DRV at high μM ranges [22]. Consequently, we sought to characterize the precursor autoprocessing mechanism by alternative approaches that avoid the *in vitro* denature-and-refold cycle. For example, we recently engineered various fusion precursors by sandwiching the p6*-PR precursor between a variety of proteins and/or epitope peptides to study precursor autoprocessing in the context of transfected mammalian cells [18,20,24,30]. These fusion precursors consistently recapitulated the autoprocessing phenotypes reported with other approaches [6,16,18,30], indicating a similar underlying mechanism and validating the use of this assay as a simple model for autoprocessing studies. Here, we present results from this model system, a standard proviral expression system, and covariance analysis to analyze the roles of residues 77 and 93, which are distant from each other according the mature protease structure, as they relate to precursor autoprocessing.

Covariance analysis refers to the identification of amino acid pairs that vary together across independent genetic sequences. Covariance can arise from selective pressures that imply a coordinated function for the involved pairs, or from a chance linkage resulting from a genetic bottleneck. Analysis of a wide range of viral genomes recently revealed that the large majority of amino acid covariances in viral genomes form genome-wide networks of coordinated variation [31]. These networks appear to arise through selection, because the sequence sets were monophyletic and control analyses seeking to model the networks though a genetic bottleneck failed to generate networks similar to the ones found in nature. Covariance analysis has also been used to identify residue pairs that directly interact as close contacts in their 3-dimensional structures [32,33]. For example, covariation analyses in nucleotide sequences have been successfully applied to construct accurate models of tertiary structures of self-splicing RNAs [34,35]. Genome-wide networks of amino acid covariances have been used to evaluate viral evolutionary patterns in Hepatitis C Virus (HCV) [36], and patterns in the HCV covariance networks are associated with response efficacy to IFN-α-based therapy for HCV patients [37–39].

Amino acid covariation analysis has previously been used to analyze the development and patterns of HIV-1 mature PR and reverse transcriptase drug resistance mutations in the presence of antiretroviral therapy [40–42]. In this study, we applied a covariance analysis to compare and contrast p6*-PR and mature PR structural topology in order to gain insights into the precursor autoprocessing mechanism. Our analysis confirmed previously identified covariance pairs in the mature PR region and found additional covariance pairs that suggest intricate interactions between the p6* peptide and PR, which led to the development of the first precursor PR topological model. We further examined covariance between residues 77 and 93 using our autoprocessing model and revealed that a functional interaction between these two residues differentially modulates precursor autoprocessing and mature protease activity.

## Materials and Methods

### Sequence collection and analysis

We initially collected 278 HIV Subtype B amino acid sequences containing the Gag-Pol region from the online LANL HIV Sequence Database (http://www.hiv.lanl.gov). These sequences are publically accessible and are not linked to any patient identification information. Sequences with an incomplete p6*-PR region and those not documented as drug-naïve were discarded. The remaining 219 sequences were edited to include only the p6*-PR-RT region. Sequences were aligned and analyzed via bootstrap phylogeny (1000 replicates) using the neighbor-joining method. Included in the alignment were reference sequences from the HIV-1 subtypes A, B, C, F, H, J, K and U along with reference sequences from Groups N, O, and P. This phylogenetic analysis was conducted to ensure that each sequence represented an independent viral isolate and was representative of subtype B. Sequences that grouped with any of the non-B subtypes were discarded. Subsequently, the final number of sequences used for covariance analysis was 147. Phylogenetic and molecular evolutionary analyses were conducted using *MEGA* version 4 [43].

### Sequence alignments and covariance identification

Alignments for use in the covariance algorithm were generated using MUSCLE [44]. Covariant positions in the sequence alignments were identified by applying the observed-minus-expected-squared (OMES) approach to all possible pairs of amino acid positions using our previously described methods [37]. To identify the covariance pairs, we calculated for every possible pair of columns i and j, a score S using observed and expected pairs: S = (Σ^L^_l_(*N*_*OBS*_—*N*_*EXP*_)^2^)/*N*_*valid*_, where L is the list of all observed pairs and N_*OBS*_ is the number of occurrences for a pair of residues. The expected number for the pair is given by: *N*_*EXP*_ = (C_x*i*_C_y*j*_)/*N*_*valid*_, where *N*_*valid*_ is the number of sequences in the alignment that are non-gap residues; C_xi_ is the observed number of residue *x* at position *i*, and C_yj_ is the observed number of residues *y* at position *j*. The expected number of column pairs calculated in this manner provides a null model for comparisons of the observed pairs.

**Fig 2:**
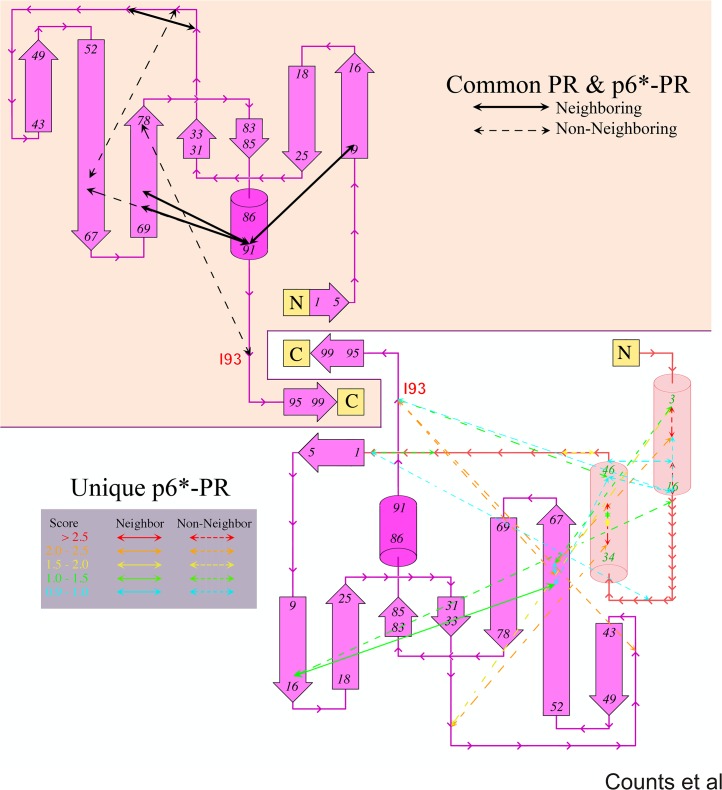
Structural topology comparison between mature PR and p6*-PR precursor. The topology map of mature PR (overlaid on a pale orange background) was constructed from information on secondary structures annotated for the single-crystal structure of the mature PR (PDB code 3DKJ). Helices are indicated by cylinders and β-strands by arrowed blocks. Covariant pairs within the mature PR that are previously identified by Wu et al and common to those identified in this study (see Tables 1 and 2) are mapped onto the structural elements as double arrowed lines (upper left). Solid lines connect covariance pairs in close spatial proximity based on the mature PR structure (neighboring), while dashed lines those not in close spatial proximity (non-neighboring). The topology map for the precursor p6*-PR (lower right) is built upon the top 25 (out of 83 with S score > 0.9) covariance pairs identified in this current study with the p6* region colored in pale red. In the lower half, solid lines connect covariant pairs that are in close spatial proximity based on the mature PR structure (neighbor), while dashed arrows indicate those within PR but not in close proximity, or within the p6* domain, and from the p6* to PR domains (non-neighbor). Only pairs unique to this study are shown.

To determine the cutoff score for S to use for each alignment, the number of covariance pairs was plotted over a range of scores. This curve was compared to a similar curve generated from alignments of sequences in which the residues at positions of variance were shuffled, and the score cutoff at which the number of covariance pairs in the shuffled alignment was ≤1% of the number of covariance pairs in the unshuffled alignment was used to define the covariances. This analysis identified 83 covariant residue pairs, 18 of which occur between PR residues. To determine the relevance of these pairs to our goal of studying precursor PR autoprocessing, we located each pair of residues on the mature PR structure using PDB structure 3DJK in *PyMol* (http://www.pymol.org). Residue pairs in close proximity to each other on the structure of the mature PR were discarded as they likely interact with each other to stabilize the mature PR conformation [32,33]. Rather, we sought pairs located at a great enough distance (>12 Å) thinking that they might be close to each other in alternative conformations that are different from the mature protease or might be functionally linked via long-range interactions. The 77–93 covariance pair was selected for further investigation because of the large distance (20.7 Å) separating the Cαs of these two residues in the mature PR structure (Fig 2C) and the relatively high covariance score of 0.93 associated with this pair.

### DNA Mutagenesis

**Fig 3:**
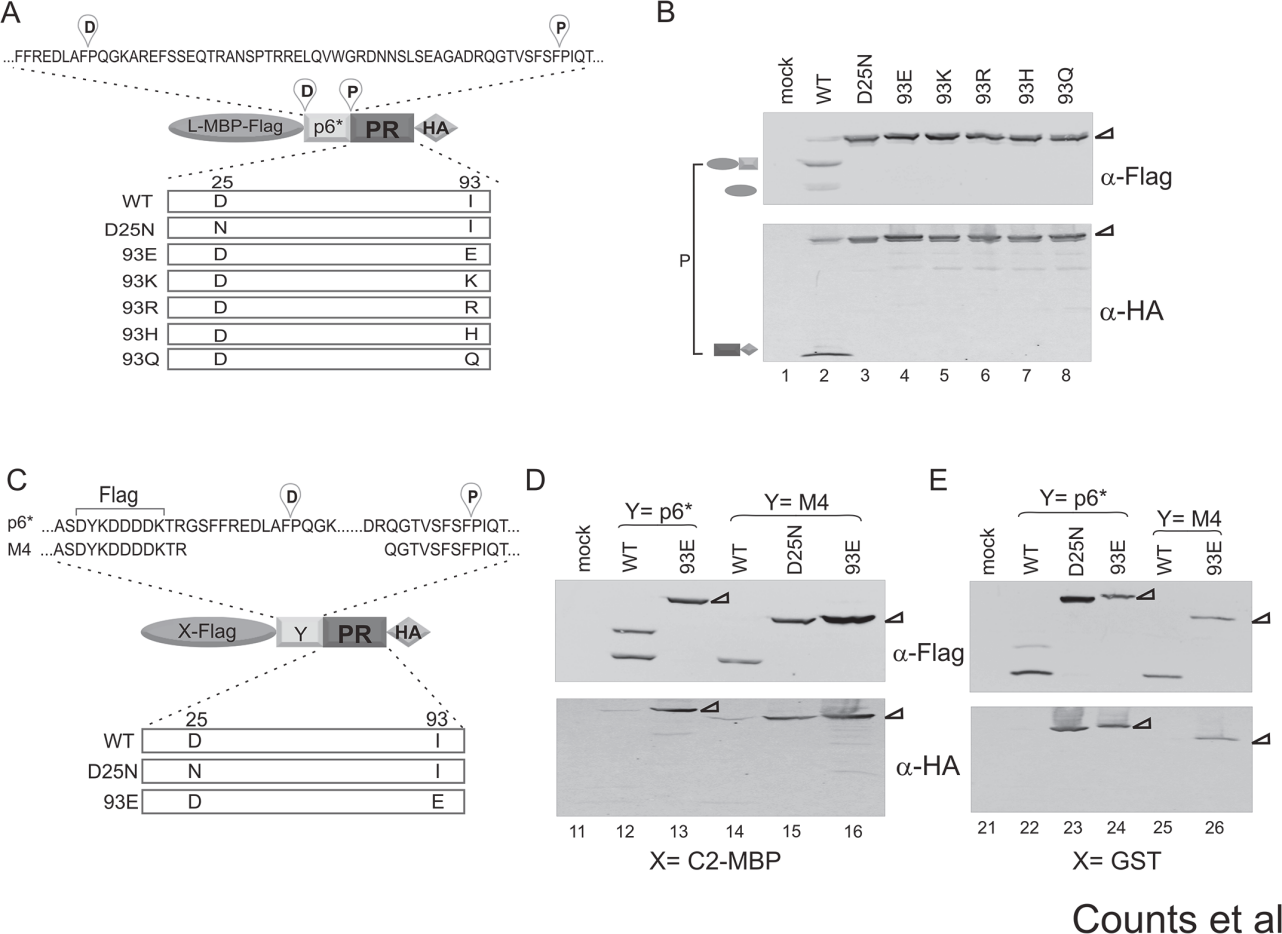
Mutations of residue 93 to non-covariance amino acids abolish fusion precursor autoprocessing. (A) Miniprecursor fusion schematic showing the distal and proximal cleavage sites and residue 93 mutants that were tested here. (B) Autoprocessing of the L-MBP fusion precursors carrying the indicated mutations. Post-nuclear total lysates were resolved on SDS-PAGE and analyzed via western blotting. Two sets of lysate samples were resolved and probed separately with mouse Flag (top) or HA (bottom) antibodies. Both were probed with IR800 goat anti-mouse secondary antibody and scanned using a LI-COR Odyssey scanner. The bracket to the left indicates the autoprocessing products resulted from the proximal site cleavage. (C) Precursor fusion schematic showing the p6* sequence and the truncated sequence of M4, in which the distal cleavage site is deleted. *X* corresponds to either C2-MBP or GST. Autoprocessing of the indicated constructs is shown in the context of C2-MBP (D) or GST (E) with the wild type or M4 p6* peptide. The corresponding full length precursors were denoted with triangles.

DNA plasmids encoding for the fusion precursors used in this study were constructed by standard mutagenesis and molecular cloning techniques as described previously [18,30]. The GST-Flag-p6*-PR-HA encoding plasmid was used as the parental expression vector [18,24]. The GST coding sequence was then replaced with the L-MBP or C2-MBP coding sequence to express fusion precursors carrying different N-terminal tags. The L-MBP tag contains the full-length maltose binding protein sequence plus an additional peptide (MATSSHHHHHHSSGLVPRGSH) fused to its N-terminus. The C2-MBP sequence is derived from the full-length MBP but lacking the N-terminal signal peptide (MKIKTGARILALSALTTMMFSASALA). Therefore, the L-MBP and C2-MBP differ only at their N-terminal sequences (C2-MBP is 46 aa shorter) and have the same C-terminal sequences (366 aa). Unless noted elsewhere, these fusion constructs contain the entire p6* sequence carrying two natural cleavage sites, one between p6* and PR (the proximal site, also referred to as the N-terminal cleavage site) and the other at the N-terminal region of the p6* peptide (the distal site) (Fig 3A). The majority of the p6* peptide was truncated in M4 construct (Fig 3C) [18]. The pNL-PR proviral construct was previously described [30]. The individual point mutations described herein were introduced to the indicated plasmids by standard PCR-mediated mutagenesis and cloning procedures. All plasmids were sequenced to confirm the presence of the desired mutations and detailed sequence information is available upon request.

### Mammalian cell culture and transfection

HEK293T cells were maintained in DMEM medium with penicillin/streptomycin and 10% fetal bovine serum (FBS). Calcium phosphate transfection was carried out as described previously [18,30]. In brief, cells were plated in 6-well plates to obtain 40–50% cell confluence at the time of transfection. About 16 h after plating, chloroquine stock solution (25 mM in water) was added to each well to a final concentration of 25μM. A total of 1μg plasmid DNA was combined with ddH_2_O to a total volume of 131.4 μL, which was then mixed with 18.6 μL of 2M CaCl_2_ by a gentle vortex. Then, 150 μL of 2x HEPES-buffered saline (HBS) (50 mM Hepes, 10 mM KCl, 12 mM Dextrose, 280 mM NaCL, 1.5 mM Na_2_HPO_4_, pH 7.04~7.05) was added drop wise to the mixture of DNA, ddH_2_O and CaCl_2_ while simultaneously mixing gently with a vortex. The final 300 μL mixture was added to each well within 2 minutes of the addition of HBS. About 8–9 hours post-transfection, the medium in each well was replaced with 3mL of chloroquine-free equilibrated DMEM medium with FBS and penicillin/streptomycin. After ~24 hours of incubation at 37° C and 5% CO_2_ post-transfection, cells were washed once with PBS and lysed using 100 μL Lysis Buffer A (25 mM Tris-HCl, pH8.0, 150mM NaCl, 1% sodium deoxycholate, 1% Triton X-100, and protease inhibitor). Lysates were then centrifuged at 20,800 x *g* for 2 minutes to pellet nuclei and other insoluble components. Post-nuclear cytoplasmic lysates were then separated into 3 aliquots and stored at -20° C or resolved immediately on SDS-PAGE.

Virus-like particles (VLPs) released from the cells were examined by collecting culture medium at ~48 hours post-transfection and a subsequent centrifugation (20,800 x *g* for 2 minutes) at room temperature to remove cell debris. The supernatant was then transferred to a clean tube with 200 μL 20% sucrose solution made in 1xPBS and centrifuged at 20,800 x *g* for 2 hours at 4°C to pellet the VLPs. Pellets were resuspended in 40 μL 1xPBS aliquots and 20 μL aliquots were subjected to SDS-PAGE.

### Western blot analysis and quantification

Post-nuclear total lysates were resolved on 12% SDS-PAGE gels followed by transfer of protein from the gel to PVDF membrane for Western blotting. Each set of samples was analyzed in duplicate to allow for separate antibody probing. Membranes were blocked by shaking at room temperature in PBST (1xPBS with 0.04% Tween-20) with 5% Fetal Bovine Serum and 0.5% gelatin for 30 minutes to 1 hour. Membranes were then separately probed with either monoclonal mouse anti-Flag or monoclonal mouse anti-HA antibody in blocking solution by shaking for 1 hour at room temperature or overnight at 4° C. Membranes were then washed in PBST three times for 5 minutes each. A goat anti-mouse IR800 secondary antibody was diluted 1:20,000 in blocking solution with 0.01% SDS and membranes were washed in this solution for 1 hour at room temperature. Membranes were then washed three times for 5 minutes each with PBST and then rinsed and stored in PBS.

Membranes were scanned using a LI-COR Odyssey infrared scanner. Images were acquired using Odyssey 3.0 or Image Studio 2.1 software. The amounts of full length miniprecursor, proximal cleavage product (L-MBP-Flag-p6*), and distal cleavage product (L-MBP-Flag) on the anti-Flag membranes were quantified in Image Studio 2.1 by selecting the bands of interest and calculating the signal of the band using Signal = Total—(Background x Area), where total is the sum of the individual pixel intensity values for the entire shape and background is the average intensity value of the pixels in the background. The percentage of full length precursor was then calculated by dividing the full length precursor signal value by total signal value (= the full length precursor signal value + the proximal cleavage product signal value + the distal cleavage product signal value). Autoprocessing efficiency was then calculated by subtracting the full length precursor percentage from 100%.

## Results

### A covariance network and a topological model of the p6*-PR miniprecursor from HIV-1 subtype B sequences

We gathered 147 drug naïve, subtype B p6*-PR (miniprecursor) sequences from the online Los Alamos National Laboratory HIV Sequence Database (http://www.hiv.lanl.gov). Note that the number of p6*-PR containing sequences in the LANL database is significantly smaller than the number of PR alone sequences. Multiple sequencing readings (mostly identical) from one patient were counted as one entry. Therefore, these 147 sequences actually represent >500 independent sequences. Phylogenetic analysis of these sequences confirmed that they are independent isolates (no duplicates) from one subtype with no deep phylogenetic splits (S1 Fig). Covariance analysis using the OMES (observed minus expected) algorithm of these sequences revealed a set of covariances that formed a single, fully connected network with hub-and-spoke typology consisting of 83 pairs in 39 nodes (Fig 1B). The cutoff S score of 0.5 was determined by comparing alignments of the same sequences in which the residues at positions of variance were randomly shuffled when the number of covariance pairs in the shuffled alignment was when the number of covariance pairs in the shuffled alignment was ≤1% of the number of covariance pairs in the unshuffled alignment (see Materials and Methods). The S scores of these pairs are between 0.500 and 4.469.

We categorized these covariance pairs into three groups based on the location of the involved residues. There are 39 pairs within the p6* region, 26 pairs between p6* and PR, and 18 pairs within the PR region (S1 Table). We first examined the 18 PR/PR pairs and compared them with a previous report [42] to evaluate the accountability of our analysis. From 1004 untreated isolates (multiple sequences for each patient), Wu et al identified 23 statistically significant PR/PR pairwise correlations. However, we were unable to compare our pairs to these correlations as they were not reported. Instead, we compared our 18 PR/PR pairs to the correlations identified from 1240 drug-experienced isolates with the assumption that covariance pairs/correlations important for both drug-naïve and drug-experienced PR should overlap between the two lists. Out of the 115 statistically significant correlations, Wu et al reported the 43 most strongly correlated pairs (the top 37%) [42]. We constructed a covariance relational map (analogous to a structural contact map) using these 43 correlations in order to visually analyze the patterns of functionally or structurally associated amino acid pairs (S2 Fig). This type of map is symmetric along the diagonal and, therefore, it can be used to identify associated pairs within each PR and between the two analyses. We found that 7 out of our 18 PR/PR pairs (7/18 = 38.9%) are either identical (2 positive pairs and 1 negative pair) or similar (4 pairs with the involving amino acids within 4 residues in sequence between the two analyses) to the previously reported correlations (S2 Fig). Furthermore, the ranking of our pairs by S value are in general agreement with the ranking of the overlapping correlations by Phi coefficient (Tables 1 and 2).

**Table 1:** PR/PR covariance pairs in close proximity per the mature protease structure.

Pos 1	Pos 2	Phi		Score	Pos 1	Pos 2	
**Positive:**							
82	54	0.63					
32	47	0.51					
90	73	0.47					
36	35	0.45					
20	36	0.41					
30	88	0.4					
90	71	0.38					
90	10	0.35					
82	10	0.35					
84	10	0.3					
84	90	0.3					
48	54	0.29					
12	19	0.29					
84	73	0.28					
82	24	0.26		1.095	15	62	
35	37	0.26		1.02	35	37	
82	48	0.25		0.955	63	64	
84	71	0.25		0.876	71	93	
90	93	0.22		0.856	62	63	
10	93	0.22		0.856	15	77	
46	55	0.22		0.665	16	39	
12	14	0.22		0.644	12	93	
10	24	0.22		0.604	15	63	
30	75	0.22					
54	20	0.22					
**Negative:**							
36	77	-0.34	Not I/I	0.735	36	77	not I/I

The left half lists the covariance pairs identified by Wu et al [42] and the right half lists those identified in this study. They are ranked by either Phi coefficient (left) or S score (right).

**Table 2:** PR/PR covariance pairs NOT in close proximity per the mature protease structure (3DJK).

Pos 1	Pos 2	Phi	Score	Pos 1	Pos 2
54	10	0.41			
71	10	0.37	1.8	41	93
46	10	0.35	1.68	62	93
71	54	0.34			
77	93	0.31	0.98	77	93
46	24	0.27			
10	73	0.27	0.87	41	77
82	71	0.26			
54	24	0.26			
36	62	0.26	0.79	35	63
82	46	0.25			
90	63	0.23	0.7	93	63
90	20	0.22	0.64	41	64
54	20	0.22	0.55	14	37
48	10	0.21			

The left half lists the covariance pairs identified by Wu et al [42] and the right half lists those identified in this study. They are ranked by either Phi coefficient (left) or S score (right).

We next constructed a precursor topology map using the identified p6*/p6* and p6*/PR covariance pairs (Fig 2). The relational map of p6*-PR also provides clues to the structure of the N-terminal p6* domain in the context of the precursor (S2 Fig). A set of regularly spaced covariance pairs near the main diagonal suggests that there are two potential α-helices (residues 5 to 17 and 36 to 48 of p6* peptide). These two helices show a significant number of covariance pairs among them and to each the N- and C-terminal end of the mature PR protein. These connections suggests that the two p6* helices are aligned to help mediate interactions between the two sequence ends of the precursor and the mature PR. A closer inspection of the trends suggests that helix I is connected to the C-terminus, helix II is connected to the N-terminus, and mediation occurs through strand 52/67 (PR residues 62–64 showed multiple connections to these p6* helices). The relational and associated topology map suggest that the p6* domain may interact with the protease domain and modulate precursor autoprocessing. This will provide guidance for the study of precursor structure and function in the future.

The 18 PR/PR covariance pairs were further divided into two subgroups according to the distance (alpha carbon to alpha carbon) between the involved residues in a mature PR structure (3DJK). We defined pairs with < 12 Å distance as covariance pairs in a close proximity (10 out of 18) and those with >12 Å distance as covariance pairs not in close proximity (8 out of 18). The 12 Å cut-off value was an estimated maximum distance at which two residues may directly interact with each other. This analysis was similar to what was reported by Wu el al, in which residues within 8 Å of each other (the shortest interatomic distance between any atoms in the two residues) were considered to be neighboring pairs [42]. The short-distance (neighboring) PR/PR pairs are likely involved in maintaining/stabilizing the mature PR structure. Thus, these short-distance pairs conform to the three-dimensional structure of the mature PR protein. Out of the eight long-range (non-neighboring) PR/PR covariance pairs, four of them (1 identical and three similar) were reported previously and matched well in their ranking orders (Table 2). Considering the size and topology of HIV-1 mature PR, these long-distance correlations are statistically unlikely to be chained covariations [42]. Therefore, the biological significance of these covariance pairs remains largely elusive. To further evaluate the biological significance of these covarying pairs, we focused our effort on examining the role of 77–93 covariance in precursor autoprocessing because both Wu et al and our OMES analysis independently identified this same pair with high statistical significance.

### Sequence variations and preference of residues 77 and 93

Among the 147 sequences used for covariance analysis, residue 93 has alkyl amino acid I in 68.7% of sequences and the L variant accounts for 30.6%, with one sequence carrying M. At position 77, the predominant amino acid is V (74.8%) followed by I (25.2%) (S1 Fig). These variations give rise to five combinations of 77–93 pairs, based on which we calculated their distribution preferences by *chi*-squared analysis (Table 3). Interestingly, when position 93 is the most common I, position 77 showed a preference of V over I. However, when position 93 has the less prevalent L, position 77 displayed a reversed preference, *i*.*e*., 77I is favored over 77V. With M at position 93, 77I is also favored over 77V. These distinct residue 77 preferences suggested a functional interplay with residue 93, although the *chi*-squared analysis was not statistically significant (p = 0.12), likely due to a small sample size (n = 147).

**Table 3:** Distribution of 77–93 covariance pairs.

77–93 Pair	Observed	Expected	Preference	p-value
V-I	83	75.7	Favored	
I-I	18	25.2	Disfavored	
V-L	28	33.7	Disfavored	0.12
I-L	17	11.4	Favored	
V-M	0	0.7	Disfavored	
I-M	1	0.3	Favored	

This is derived from the 147 sequences we used for covariance analysis.

We postulated that if a functional interplay truly occurs between residues 77 and 93, a larger number of samples would demonstrate the same trend with greater statistical significance. We randomly collected 322 drug naïve, subtype B PR sequences from the Stanford University HIV Drug Resistance Database [45,46] and performed the same pair preference analysis (Table 4). The same association was observed with a p-value of < 0.001. Such context-dependent preferences between residues 77 and 93 further suggested a functional link between them.

**Table 4:** Distribution of 77–93 pairs of an independent set of 322 PR Sequences.

77–93 Pair	Observed	Expected	Preference	p-value
V-I	209	193.2	Favored	
I-I	34	49.8	Disfavored	
V-L	33	45.3	Disfavored	
I-L	24	11.7	Favored	<0.01
V-M	8	9.5	Disfavored	
I-M	4	2.5	Favored	
V-V	6	8	Disfavored	
I-V	4	2	Favored	

This is derived from 322 drug naïve, subtype B sequences collected from the Stanford University HIV Drug Resistance Database.

### Alterations of I93 to non-covariant amino acids abolish precursor autoprocessing

Residue 93 was previously categorized as an accessory residue as it is buried within a hydrophobic environment that is far away from the catalytic site and substrate-binding groove [42]. In order to evaluate its potential interaction with residue 77, we first determined if a polar or charged residue could be placed in this position for complementation testing. We used a cell-based precursor autoprocessing assay that allows for the examination of precursor autoprocessing in transfected HEK293T cells [18]. The prototypical fusion precursor consists of a p6*-PR minprecursor sandwiched between GST and a small peptide epitope such as HA. GST was initially chosen to facilitate precursor dimerization, which is believed to be important for precursor autoprocessing. The dissociation constant for dimeric GST is in the low nM range [47] and the GST C-termini in the dimer (PDB 3KMN) are also in a close proximity to help bring the precursor protease together. We later observed that fusion precursors carrying tags such as GFP, hsp70, and MBP in the place of GST are autoprocessing competent as well ([20] and unpublished data). In this study, we mainly used fusion precursors that have an L-MBP fusion tag (see materials and methods for sequence) upstream of the p6*-PR miniprecursor, as the mature PR released from these L-MBP fusion precursors is readily detectable. The wild type p6* peptide contains two native cleavage sites (Fig 3A) and precursor autoprocessing at these sites releases distinct products. Our previous report demonstrates that these two cleavage reactions are relatively independent of each other [20], which serves as an internal control to simultaneously examine the cleavage reactions at both sites in the transfected cells.

Five amino acid substitutions were introduced at residue 93 in the context of L-MBP fusion precursor (Fig 3A). The wild type L-MBP fusion precursor undergoes effective autoprocessing in transfected cells, releasing several products detectable by western blotting (Fig 3B, lane 2). Precursor autoprocessing at the proximal site releases L-MBP-Flag-p6* (Fig 3B, the anti-Flag blot) and the HA-tagged mature PR (Fig 3B, the anti-HA blot). Precursor autoprocessing at the distal site releases L-MBP-Flag and p6*-PR-HA, but this latter fragment is not detectable due to rapid self-degradation, as previously reported [18,20]. The catalytic site mutation D25N completely abolishes autocatalytic activity as evidenced by the accumulation of full length precursor and the lack of any autoprocessing product (Fig 3B, lane 3). Mutations I93E, I93K, I93R, I93H, and I93Q also abolished precursor autoprocessing, displaying a detection pattern very similar to that of the D25N mutation (Fig 3B, lanes 4–8). We cannot discern yet whether these residues cause misfolding of the mature PR or directly disrupt interactions among covarying pairs. Nonetheless, our data confirm that position 93 will accept branched non-polar amino acids, but a substitution with polar or charged residues does not support precursor autoprocessing activity.

To further verify that the observed autoprocessing inhibition was not an artifact of the L-MBP fusion precursor, we introduced the I93E mutation into four other fusion constructs to examine the effects of this mutation in various contexts (Fig 3C). The I93E mutation in the context of C2-MBP or GST fusion also demonstrated complete inhibition of precursor. This remains true in the presence of either the full-length p6* peptide or M4, a truncated version of p6* in which the N-terminal 48 amino acids were deleted (Fig 3D&3E). Collectively, our data illustrated that although a few variances are found at residue 93, alterations to charged or polar amino acids severely impede precursor autoprocessing.

### Changing V77 to non-covariant amino acids also impedes precursor autoprocessing

**Fig 4:**
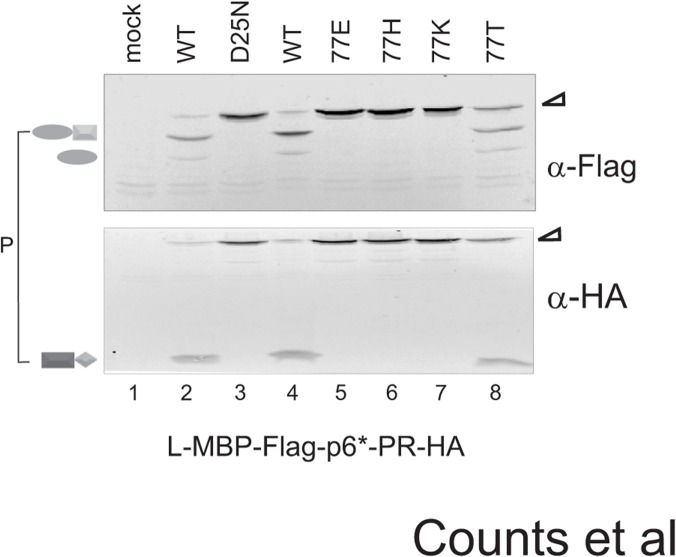
Mutations of residue 77 to non-covariance amino acids abolish fusion precursor autoprocessing. Autoprocessing of L-MBP fusion precursors carrying the indicated 77 mutations in transfected HEK293T cells. The bracket indicates the proximal cleavage products.

We next examined amino acid substitutions at position 77 (Fig 4). Alterations of charged amino acids (E, H, and K) completely inhibited autoprocessing in a manner similar to that of D25N (Fig 4, lanes 3, 5–7). A construct expressing a V77T mutation showed partial autoprocessing activity as it produced HA-tagged mature PR (Fig 4, lane 8) along with some unprocessed full-length precursor. This suggests that some polar residues at position 77 do not completely abolish precursor autoprocessing. Nonetheless, our data illustrated a limited acceptance of diverse amino acids at residue 77 that will support normal precursor autoprocessing.

### Residues 77 and 93 have distinct preferences for the alkyl amino acids

**Fig 5:**
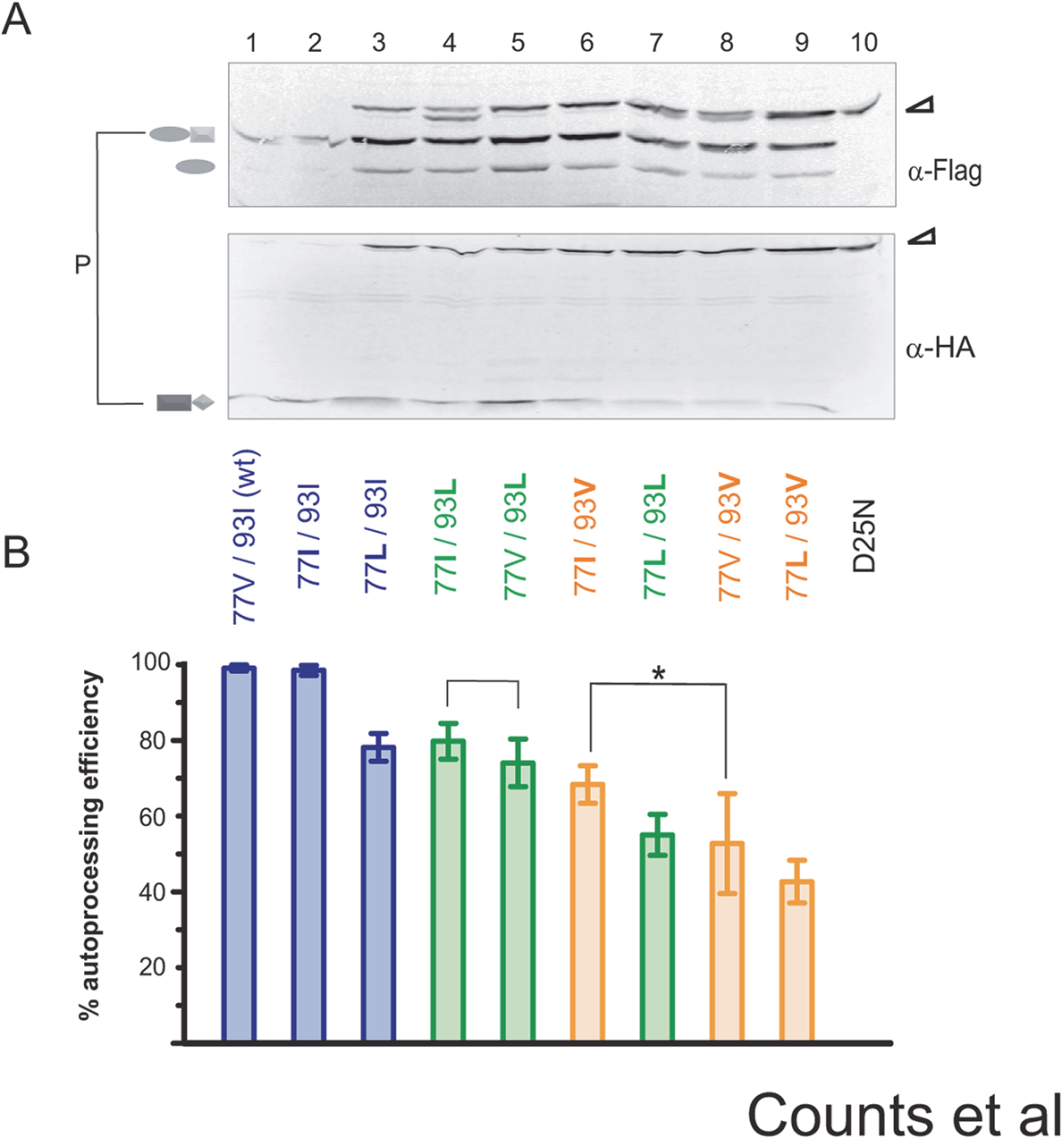
Precursor autoprocessing of various 77–93 covariance pairs. Autoprocessing (A) and quantification (B) of L-MBP fusion precursors carrying the indicated variances in transfected HEK293T cells. Autoprocessing efficiency was determined by quantifying the amounts of L-MBP-Flag-p6* and L-MBP-Flag (the proximal and distal cleavage products) as a percentage of the sum of those products plus the full-length unprocessed precursor detected by Flag antibody in each lane. Constructs are arranged in the order of decreasing autoprocessing efficiency from left to right. Brackets indicate an increase in autoprocessing efficiency observed when a V77I mutation is combined with I93L and I93V mutations; the asterisk denotes statistical significance (p = 0.0162). Quantification values are based on at least 4 independent biological samples, and error bars represent standard deviations.

Because individual substitutions of charged or polar amino acids at positions 77 and 93 abolish precursor autoprocessing, we didn’t examine mutations with oppositely charged residues placed at these two positions for complementation. Instead, we postulated that the distribution of naturally occurring variants at sites 77 and 93 would be positively correlated with autoprocessing activity, *i*.*e*., higher autoprocessing efficiency would be observed with more commonly occurring variances. Subsequently, we engineered fusion precursors carrying variations (V, I, or L), individually at each position or in combination, in the context of the L-MBP fusion backbone to test this thought. A monoclonal Flag antibody was used to detect the full-length precursor, proximal cleavage product (L-MBP-Flag-p6*), and distal cleavage product (L-MBP-Flag) in each lane. Band intensity was quantified to represent the amount of product each band represents. This allowed us to directly examine the fusion precursor and its autoprocessing products at the steady state in the cell lysate. We defined autoprocessing efficiency as the percentage of released products relative to the sum of the full-length precursor plus the released products. Accordingly, high autoprocessing efficiencies correspond to high percentages of released products, while low autoprocessing efficiencies correspond to low percentages of release products and accumulation of full length precursors (Fig 5). We did not quantify PR-containing products for autoprocessing efficiency analysis as these fragments are known to undergo self-degradation [18,20,48] and thus would compromise data interpretation.

Our data demonstrated that variances at residue 93 play an important role in determining autoprocessing efficiency in the context of the L-MBP fusion precursor. In fact, the constructs can be roughly divided into three tiers (color coded in Fig 5) based on their autoprocessing efficiencies. Tier 1 consists of the constructs carrying the most-observed 93I, tier 2 constructs have 93L, and tier 3 constructs have 93V. We therefore concluded that position 93 has defined preferences out of three tested variants (I>L>V) with the most frequently occurring I associated with the highest efficiency of precursor autoprocessing. All three amino acid variants are hydrophobic with similar structures, suggesting that even minor amino acid changes at this position impact precursor autoprocessing activity.

Variations at residue 77 also modulate precursor autoprocessing efficiencies. The most frequently observed residue at position 77 is V followed by I, while L is rarely observed (S1 Fig). Within each residue 93 tier, the 77L variant always showed the lowest autoprocessing efficiency, suggesting that L at position 77 is least supportive of precursor autoprocessing. This is consistent with its very low naturally occurring frequency. A notable finding is that within the second and third tiers (93L and 93V), 77I consistently showed higher autoprocessing efficiency than 77V even though 77I is less prevalent than 77V in drug naïve sequences. For example, in the context of 93L (the second tier), 77I displayed higher autoprocessing efficiency (80%) than 77V (74%). It also had an extra cleavage product that is slightly smaller than the full-length precursor (Fig 5A, lane 4). In the context of 93V (the third tier), 77I also showed higher autoprocessing efficiency (68%) than 77V (53%) with statistical significance (p = 0.01 by t-test). Little difference in autoprocessing efficiency was observed between 77I and 77V in the context of the wild type 93I (the first tier). We interpreted that the wild type 93I already conferred the full autoprocessing activity in this model system and thus additional enhancement conferred by 77I, if any, is undetectable. Collectively, this analysis revealed that residue 93 prefers I>L>V in supporting precursor autoprocessing; residue 77 prefers V>I>L in the context of the 93I but prefers I>V in the context of other 93 variances.

### A functional interplay between residues 77 and 93

**Fig 6:**
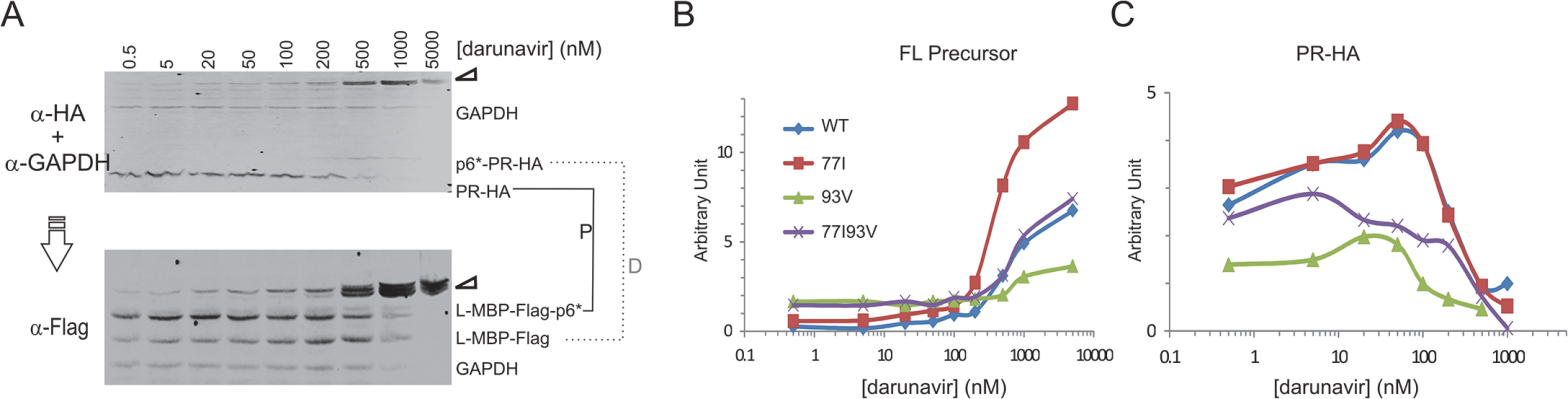
Different 77–93 variances respond to increasing darunavir differently. (A) Western blot analysis of the wild type L-MBP fusion precursor in response to increasing concentrations of darunavir. Transfected HEK293T cells were treated with the indicated concentrations of darunavir for ~15 h. The post-nuclear total lysates were then collected and resolved on SDS-PAGE. The resulting blot was first analyzed with HA and GAPDH antibodies followed by visualization by IR800 secondary antibody (upper image). The same blot was then re-probed with a Flag antibody to detect Flag-containing processing products (lower image). The full length precursor is indicated by triangles on the right. The proximal (P) and distal (D) cleavage products are connected by a solid and dashed line, respectively. Western blots of the other three precursors are shown in S3 Fig. The amount of the full length precursor (B) or the released mature PR (C) was determined by band intensity normalized to the GAPDH intensity in each lane and plotted as an arbitrary unit (AU) over darunavir concentrations.

Our data in the previous section demonstrated that 77I conveys a higher precursor autoprocessing activity than 77V when residue 93 is L or V. This is the first biological evidence supporting the idea that there is a functional interplay between residues 77 and 93, *i*.*e*., 77V is preferred by 93I whereas 77I is preferred by 93L or 93V in supporting precursor autoprocessing. These results also align well with the residue distribution preferences observed with the 77–93 covariance pairs (Tables 3 and 4). To further examine this functional interplay, we compared the response profiles of a panel of 77–93 variants to the protease inhibitor darunavir [29] (Fig 6). Note that despite various autoprocessing efficiencies, all tested covariance pairs were autoprocessing competent. The least favorable pair (77L/93V) showed ~50% precursor autoprocessing efficiency compared to the most common pair. We therefore chose to compare 77V to 77I in the context of 93V (the 3^rd^ tier variant) as this would allow us to observe any subtle differences in the background of the low activity associated with 93V. We speculated that if these two residues functionally complement each other, the individual 77I and 93V variants would differentially impact precursor autoprocessing but the double variant would restore normal activity.

Transfected cells were treated with darunavir ranging from 0.5 to 5000 nM. At low darunavir concentrations, the most common variant (77V93I) underwent effective autoprocessing and the full length precursor was therefore not detectable. As darunavir concentration increased, precursor autoprocessing was suppressed, leading to accumulation of the full length precursor in the cell lysates. We quantified the full length precursor and the released PR-HA normalized to GAPDH as a function of darunavir concentration, respectively. This allowed us to simultaneously examine precursor autoprocessing and the released PR-HA under the same context in transfected cells. The same blot was then probed with Flag antibody to visualize other Flag-containing cleavage products (Fig 6A and S3 Fig). Assuming transfection efficiency and precursor expression level are the same among these samples, the normalized values will reflect the relative amounts of these proteins at steady state in cells. Our data support a functional complementation between residues 77 and 93 and also demonstrate that these different 77–93 variants differentially influence precursor and mature protease activities. The 77I precursor appeared to be more sensitive to darunavir inhibition as high darunavir concentrations led to accumulation of higher amounts of the full length precursor. The 93V variant reduced the overall precursor autoprocessing (Fig 5) but displayed some resistance to darunavir inhibition because high darunavir concentrations did not significantly increase accumulation of the full length precursor (Fig 6B). Interestingly, the 77I93V double variant exhibited a precursor profile very similar to the 77V93I variant (the most common one) (Fig 6B). This suggests that the 77I variant alone increases the precursor’s sensitivity to darunavir and 93V alone decreases precursor’s sensitivity to darunavir, whereas a functional interaction between the two complemented these two opposite effects.

Quantitative analysis of mature PR self-degradation in response to darunavir treatment also supports a functional interaction between residues 77 and 93. It is worth noting that the released mature PR exists in a dynamic equilibrium between its production as a result of precursor autoprocessing and its disappearance as a result of self-degradation [18,20,48]. At low darunavir concentrations (< 200 nM) when precursor autoprocessing was minimally suppressed, increasing darunavir concentration suppresses mature PR self-degradation, leading to increased detection of mature PR. When darunavir concentration further increases, inhibition of precursor autoprocessing manifests leading to the decline of mature PR production and detection. Consequently, the mature PR typically exhibits a bell-shaped detection profile over increasing darunavir concentrations (Fig 6C) [20]. Our analysis demonstrated that the 77I variant by itself retained a response profile very similar to the 77V93I control, suggesting that this single mutation had no significant effect on the mature PR self-degradation. The 93V variant by itself produced less mature PR, due to reduced autoprocessing efficiency (Fig 6B), but otherwise displayed a similar profile to the wild type control. Intriguingly, the 77I93V double variant demonstrated a distinct profile in that detection gradually declined with increasing darunavir concentration and lacked a clearly defined peak (Fig 6C). This suggests that self-degradation of 77I93V mature PR was not significantly influenced by darunavir. Consequently, decline in its detection was solely correlated with its production resulting from precursor autoprocessing. In this case, the functional interplay between 77I and 93V confers a property that is distinct from each individual variant such that self-degradation of the 77I93V mature PR becomes resistant to darunavir.

### The 77I/93V covariant displays different effects on precursor autoprocessing and mature PR activity in the context of a proviral construct

**Fig 7:**
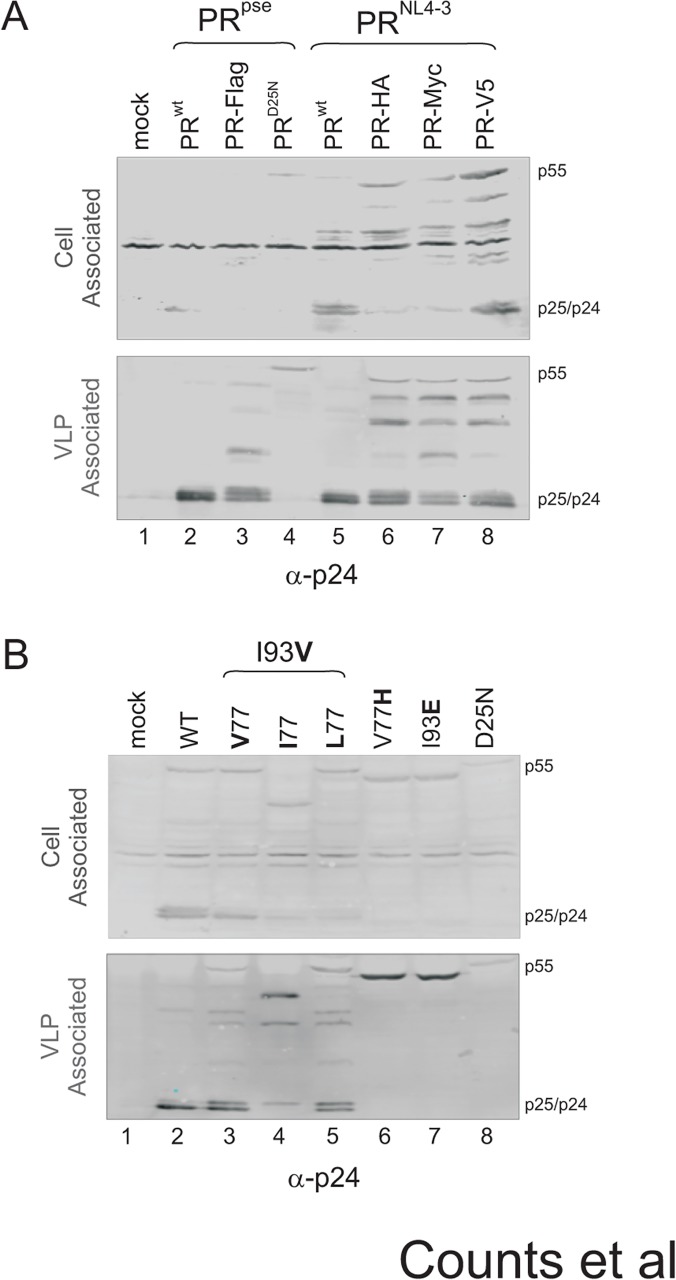
Gag processing in the presence of various C-terminal tags (A) and 77–93 covariants (B) in transfected HEK293T cells and the released virus like particles. The blots were probed with a mouse monoclonal anti-p24 antibody and visualized by a LI-COR Odyssey scanner.

We next sought to examine whether the observed functional correlation is reproducible in a proviral context. Because the fusion precursor system utilizes a tag at the C-terminus of the precursor to facilitate mature PR detection, we first examined whether various C-terminal tags would have any impact on Gag processing as an indirect readout of precursor autoprocessing and mature PR activity. A previously reported pNL-PR construct [18,30] was used to serve as a positive control, to which we introduced Flag, HA, Myc, and V5 tags individually fused to the C-terminus of PR. The resulting constructs were then expressed in transfected HEK293T cells. A mouse monoclonal p24 antibody was used to detect the full-length Gag precursor (p55) and the other p24-containing products associated with the total cell lysates and released virus-like particles (VLPs). We also tested two PR sequences, *i*.*e*., the pseudo wild type PR [15,24] and NL4-3 derived PR, which differ by six amino acids, to see if there is any difference in Gag processing by different PRs. Both wild type PR^pse^ and PR^NL4-3^ produced p24 as the dominant band along with some p25 plus minimal amounts of full length Gag and other p24-containing intermediates in the released VLPs (Fig 7A, lanes 2 and 5), confirming the presence of the mature PR as a result of effective precursor autoprocessing. As a negative control, a D25N mutant completely abolished PR activity, leading to detection of the full length Gag polyprotein (p55) as the predominant product (Fig 7A, lane 4). The fusion of Flag, HA, Myc, and V5 tags all resulted in greater levels of p25 and the detection of several p24-containing intermediates plus full length Gag in transfected cells and VLPs (Fig 7A, lanes 3, 6–8). Our data suggested that all C-terminal fusions have moderate impact on Gag polyprotein processing in the provirus context. It is not discernable whether the impact is due to effects on the mature PR, the precursor, or a combination of the two. Nonetheless, this is consistent with our previous publication [20] and previous reports showing that C-terminal fusions to RT or GFP remain replication competent but with reduced infectivity [12–14]. Interestingly, each fusion exhibited a slightly different Gag processing pattern in that certain intermediate(s) are more predominant than others. This suggests that PR autoprocessing and/or proteolysis activities can be modulated by various flanking sequences as well. In light of this information, we decided to evaluate 77–93 covariants using the tag-free pNL-PR construct (Fig 7B).

Both I93E and V77H mutations abolished precursor autoprocessing in the context of the pNL-PR construct, as was observed in the context of fusion precursors (Fig 3B, lane 4 and Fig 4, lane 6). They exhibited a Gag processing pattern very similar to that of the D25N control, with full-length Gag (p55) being the predominant product observed in both transfected cells and VLPs (Fig 7B, lanes 6 & 7). Our data confirmed that autoprocessing deficient mutants identified by the fusion precursor system are also defective at autoprocessing in the proviral context.

We then tested the 3^rd^ tier panel (*i*.*e*., 93V-containing covariances) to further examine their effects on precursor autoprocessing and mature PR activity. Note that Gag processing patterns in the released VLPs are an indirect reflection of these effects as different protease activities generate different Gag processing patterns. In particular, the mature PR is absolutely required for the production of the p24 capsid protein [16,24,30,49,50]; the p6*-PR precursor is unable to cleave p25 to generate p24. However, the precursor is capable of processing some other cleavage sites in the Gag polyprotein [16]. As shown in Fig 7B, both 77V93V and 77L93V displayed increased detection of the full length Gag polyprotein and other p24-containing intermediates including p25 compared to the wild type control (lanes 3 & 5). Given that the 3^rd^ tier 93V fusion precursors showed the lowest autoprocessing efficiencies in our model system (Fig 5B), we conclude that the reduced Gag processing was due to a reduced precursor autoprocessing that made less mature PR, leading to reduced production of p24. Meanwhile, it is possible that the 77V93V and 77L93V precursors by themselves might also have reduced activity in Gag processing. Collectively, the combination of these two factors led to less efficient Gag processing in VLPs. It is worth noting that regarding the types of the p24-containing intermediates, the overall Gag processing pattern of these two variants is very similar to those displayed by the proviruses containing C-terminal tags (Fig 7A).

The 77I93V covariant displayed a Gag processing pattern distinct from the other two covariants in that there was almost no detection of p24 in VLPs (Fig 7B, lane 4). Assuming that 77I93V does not prevent liberation of the mature PR, this suggests that the released 77I93V mature PR is deficient in processing p25 into p24. Note that the 77I93V mature PR released from L-MBP fusion precursor also demonstrated a darunavir response profile distinct from other control variances (Fig 6C). Consequently, we interpreted that the 77I93V combination altered activity of the resulting mature PR due to the interplay between these two residues. This interplay appeared to impair mature PR activity in the context of the NL4-3-derived PR sequence. Another distinction is that no accumulation of the full length Gag was observed in the 77I93V variant, which is quite different from the other 77–93 variants. This suggests that the 77I93V variant actually exhibited enhanced processing of the full length Gag protein compared to the other two variants. A third distinction is accumulation of a p24-reactive band (~45 KD in size) that was not detected in other constructs including the wild-type control. According to the apparent molecular weight, it appeared to result from a cleavage at NC/p6 from the full length Gag polyprotein. A previous study reported detection of a similarly sized intermediate in virions expressing p6*-PR precursors in the absence of mature PR activity [16]. In light of our data showing that no p24 was produced as an indication of lack of mature PR activity, we suggest that this intermediate was processed by the 77I93V precursor from the full length Gag polyprotein. Taken together, our data illustrates that the 77I93V covariant differentially regulates the precursor and mature PR in their activities. This observation is unique to the 77I93V combination, and is not observed with the 77V93V and 77L93V covariants, once again suggesting specific interplays (residue preferences) between these two positions in the regulation of precursor autoprocessing and mature PR activity.

## Discussion

We identified 83 covariant residue pairs by OMES analysis of p6*-PR precursor sequences derived from drug-naïve patients. There were more covariance pairs within the p6* region (39 pairs) or between p6* and PR (26 pairs) than within the PR region (18 pairs) (Figs 1 and 2 and S1 Table). Biochemical analyses of the 77–93 covariance pair suggest its involvement in differential regulation of precursor autoprocessing and mature PR activity.

### The role of the p6* peptide

Our covariance analysis identified many p6*-p6* covariance pairs that cluster into two predicted α-helices, one at the N-terminal region (Helix I) and the other at the C-terminal region (Helix II) of p6* (Fig 2 and S2 Fig). The coding sequences of these two helices also overlap with the RNA elements essential for Gag-Pol synthesis via ribosomal frameshift [16,49,51]. Thus, an intriguing question arises: are these helices simply translated from the conserved RNA sequences, or might they play an additional role in regulating PR autoprocessing? Our OMES data supports the latter possibility in that these helices also showed covariances with PR residues (Fig 2). Interestingly, almost half of the p6*-PR interaction pairs (12/26) are connected to a 3-amino-acid region consisting of I62/L63/I64 that is part of the longest β-strand in the folded mature PR. The bi-peptide E35/M36 of PR seems to be another ‘hot spot’ showing covariance with five p6* residues scattered throughout the p6* peptide. The last p6* residue (F56) covaries with four other p6* residues and three PR residues (R57, L63, I93) that are not within close proximity according to the mature PR structure. We suggest that the p6* peptide might contribute to modulating precursor autoprocessing through these potential interactions.

Previous results from our group and others demonstrate that majority of the p6* peptide is not required for precursor autoprocessing [18] and viral replication *in vitro* [49]. However, our OMES data demonstrate complex genetic interactions between p6* and PR, suggesting a possible role of the p6* peptide in regulating precursor autoprocessing. One hypothesis that reconciles this apparent discrepancy is that the p6* peptide might function as a negative regulator during precursor autoprocessing in the proviral context. Subsequently, removal of p6* would not impair precursor autoprocessing. Consistent with this hypothesis, Partin et al. reported more than two decades ago that deletion of the p6* peptide enhances precursor autoprocessing in vitro [52]. Such a negative regulation might not be required for the fitness of wild type viruses but might become essential under conditions that are suboptimal for productive viral replication. One such example was previously described in which an indinavir-resistant PR failed to dimerize by itself, but its activity was restored by the p6* peptide [53].

### A functional interplay between residues 77 and 93

The 77–93 covariance pair was previously reported through sequence analysis by Wu, et al [42] and was also identified by our OMES analysis. In this report, we experimentally examined this covariance pair for its biological function. At these two positions, only hydrophobic amino acids (I, V, L, and M) are observed in naturally occurring variances and our data confirm that charged or polar amino acids at these positions abolish precursor autoprocessing (Figs 3 and 4). This is not unexpected as both residues are buried in a hydrophobic environment according to the mature PR structure. An interesting observation is that each position has a distinct preference order in supporting precursor autoprocessing despite very similar structures of these hydrophobic amino acids. This suggests that even a minor amino acid change at these two positions could impact precursor autoprocessing activity. Our covariance analysis (Fig 1B) offers a possible justification in that residue 93 connects to nine other amino acids (including residue 77) and residue 77 connects to three other amino acids in addition to residue 93 (Fig 1B and S1 Table). Because of these extensive connections, these two residues are susceptible to higher selection pressure against random alterations and thus are likely sensitive to even minor alterations.

In addition to distinct preferences of hydrophobic amino acids in these two positions, both covariance and biochemical analyses provide evidence suggesting a functional interplay between residues 77 and 93. We tested all the possible combinations of I, L, and V variations at these two positions (Fig 5). All the combinations are autoprocessing competent, but there were differential preferences for residue 77 variants by different residue 93 variants, *i*.*e*., 77V is favored by 93I but 77I is preferred by other 93 variants to better support precursor autoprocessing (Fig 5). Furthermore, precursor autoprocessing profiles in response to darunavir, a PR inhibitor, demonstrated that the individual 77I and 93V variants display contrasting effects on this activity, while the 77I93V double variant shows a phenotype between the two individual variants that is also similar to that of 77V93I, the most common variation (Fig 6B). Mature PR self-degradation analysis demonstrated that the 77I93V mature PR is distinct from the other two mature PRs in its response to darunavir treatment (Fig 6C). These results suggest a functional cooperation between residues 77 and 93. This previously unrecognized interplay differentially regulates precursor autoprocessing and mature PR activity.

Given that both residues 77 and 93 also co-vary with other residues in PR and/or p6*, there is likely a multitude of other interactions that regulate HIV-1 PR proteolysis. For example, the 77I variant is often reported as signature variant associated with drug-resistant sequences [42,54–58]. According to our covariance analysis, residue 77 covaries with I15, M36, and R41 in addition to I93 (Fig 1B and S1 Table). Different combinations of these residues might provide additional mechanisms to regulate precursor autoprocessing and mature PR activity. Additionally, our analysis focused on subtype B viruses in which 93I is the predominant variant. However, in many non-B subtypes, 93L is the most common variant. By comparing the consensus sequences between these two subtypes, we observed that out of nine residues that covary with residue 93, four of them differ between the two subtypes: one shows a change in a nearby residue, and four (including residue 77) remain unchanged. We speculate that in the context of subtype B, 93L has lower activity than 93I in supporting precursor autoprocessing and mature PR activity, as demonstrated in this report (Fig 5). However, in the context of subtype C, 93L is preferred over 93I because other residues provide functions essential for effective precursor autoprocessing and mature PR activities. Therefore, our study suggests that there are likely multiple covariance combinations that modulate HIV-1 PR activity, and these combinations may or may not overlap with one another. This highlights the importance of defining the roles of individual covariance pairs in a context-dependent fashion.

### Implications of long-range covariance pairs

Our data demonstrate that residues 77 and 93 work together to differentially regulate precursor autoprocessing and mature PR activity despite being far apart from each other according to the mature PR structure (Fig 1C). One possible implication is that each residue might differently modulate catalysis, dimer formation, or overall stability; thus, the phenotype of certain covariance pairs could be different than the sum of the effects caused by single residue variants. A similar observation was previously reported with a multidrug resistant PR carrying six amino acid mutations (L10I/M46I/I54V/V82A/I84V/L90M), in which individual or subsets of mutations had minimal impact, but cooperative coupling of all these mutations conveyed drug resistance [28].

An alternative explanation could be that the precursor has a conformation different from the mature PR and that residues 77 and 93 function differently under these two contexts. This also implies conformational changes from the precursor to the mature PR during precursor autoprocessing. While we have no experimental evidence for such a conformational change, previous results from others and us support the idea. For example, the current PR inhibitors are much less effective at suppressing precursor autoprocessing than inhibiting mature PR activities; suppressing precursor autoprocessing requires low to mid micromolar concentrations of PR inhibitors, while suppressing mature PR proteolysis only requires low to mid nanomolar concentrations of PR inhibitors [15,18,20,21]. Additionally, we previously reported that a non-catalytic site mutation, H69E, abolishes precursor autoprocessing in the context of a proviral construct but only shows a mild reduction in proteolysis when tested with a recombinant mature PR [24]. This is consistent with the idea that H69E mutation prevents a conformational change essential for the liberation of the mature PR in the proviral context, whereas the recombinant H69E mature PR shows proteolysis activities similar to the wild type control when bypassing precursor autoprocessing through *E*. *coli* expression followed by *in vitro* folding. Consequently, additional structural investigation of the native precursor is critical to better understand the autoprocessing mechanism.

## Conclusions

We built the first topology map for the p6*-PR precursor using covariance pairs identified by OMES analysis of drug naïve sequences. One long-range covariance pair between residues 77 and 93 of HIV-1 PR was empirically characterized with a cell-based autoprocessing model and a NL-PR proviral construct. Both positions have small, branched hydrophobic residues (V, I, L) found naturally with a low tolerance for alterations to charged or polar amino acids. Each site has a distinct preference order for these three amino acids, with reside 93 playing a dominant role and residue 77 playing a modulating role in regulating autoprocessing activity. The most common 77V variant is preferred by the most commonly observed 93I while the 77I variant is preferred by other 93 variants (L, V, M) in supporting precursor autoprocessing, suggesting a functional interplay between these residues. The 77I93V variant increased precursor autoprocessing and Gag polyprotein processing but decreased mature PR activity, which suggests that the precursor and mature PR are differentially regulated by this covariance pair in catalytic activity, dimer formation, or structural stability.

## Supporting Information

S1 Fig Information of the 147 p6*-PR sequences used for covariance analysis in this study. Bootstrap phylogeny (A) and distribution percentages of individual variants at site 77 and 93 (B) are illustrated.
(TIF)

S2 Fig Covariance relational maps of mature PR and precursor p6*-PR HIV-1 protease. The covariance map for mature PR (lower right-hand corner) are mapped as residue-to-residue connections (residue # on both *x*- and *y*-axes, from 1–99, with the identical residue relationship along the main diagonal running from upper left-hand corner to lower right-hand corner). Lines trace the connections of covariance pairs (colored according to the Phi coefficients of Wu, et al [42]) and ending at the respective colored squares. The p6*-PR precursor covariance data are mapped on the left-hand side of the plot, with connecting lines and end point colored according to the scores defined in this study. The p6* domain and the mature PR are individually numbered as illustrated in Fig 1. Regularly spaced pairs near the main diagonal are indicative of helices, and are labeled as Helix I and II, respectively. The covariant pairs from the mature PR are transposed across the main diagonal into the PR region of the p6*-PR precursor (grey lines and squares). Pairs that are within 4 residues in sequence between the mature and precursor PR are indicated by the black circles, while pairs that are unique in this region to the precursor are indicated by red circles.
(TIF)

S3 Fig Precursor autoprocessing of three 77–93 variances in response to increasing concentrations of darunavir. Transfected HEK293T cells were treated with the indicated concentrations of darunavir for ~15 h. The post-nuclear total lysates were then collected and resolved on SDS-PAGE. The resulting blot was first analyzed with HA and GAPDH antibodies followed by visualization by IR800 secondary antibody (upper image). The same blot was then re-probed with a Flag antibody to detect Flag-containing processing products (lower image). The full length precursor is indicated by triangles on the right. The proximal (P) and distal (D) cleavage products are connected by a solid and dashed line, respectively.
(TIF)

S1 Table Covariance pairs identified by OMES analysis of 147 drug naïve p6*-PR sequences^a^. ^a^: covariance pairs are categorized into three interaction groups: p6*/p6*, p6*/PR, and PR/PR. The PR/PR covariance pairs with the involved amino acids far apart from each other per mature PR structure are highlighted in grey.
(DOCX)
