# Supplementary figures and images for: A Functional Interplay between Human Immunodeficiency Virus Type 1 Protease Residues 77 and 93 Involved in Differential Regulation of Precursor Autoprocessing and Mature Protease Activity
Source: PLoS One. 2015 Apr 20;10(4):e0123561. doi: 10.1371/journal.pone.0123561 (PMC4404164; doi:10.1371/journal.pone.0123561)

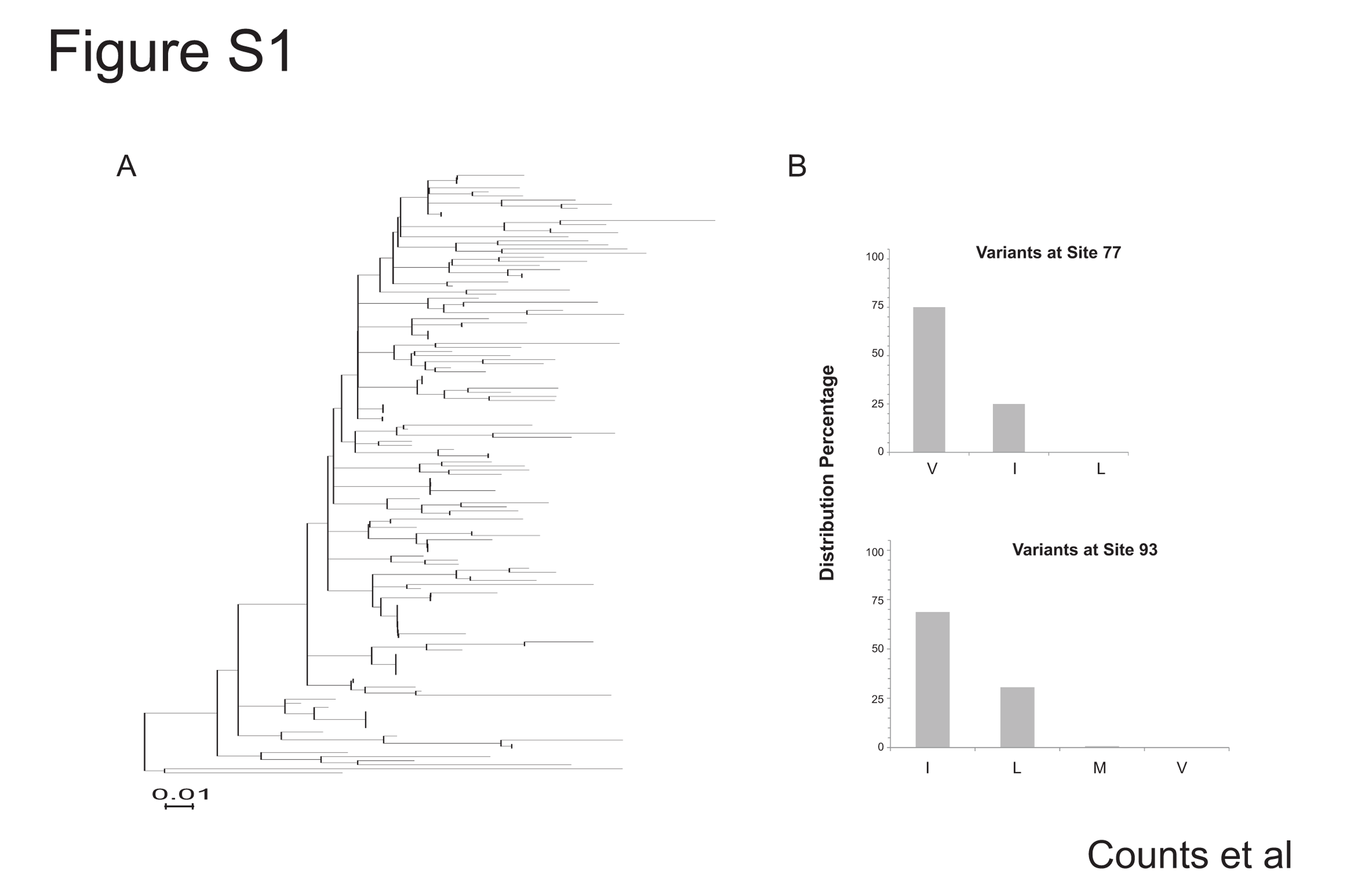

Supplement: S1 Fig — Bootstrap phylogeny (A) and distribution percentages of individual variants at site 77 and 93 (B) are illustrated. (TIF) [file pone.0123561.s001.tif]

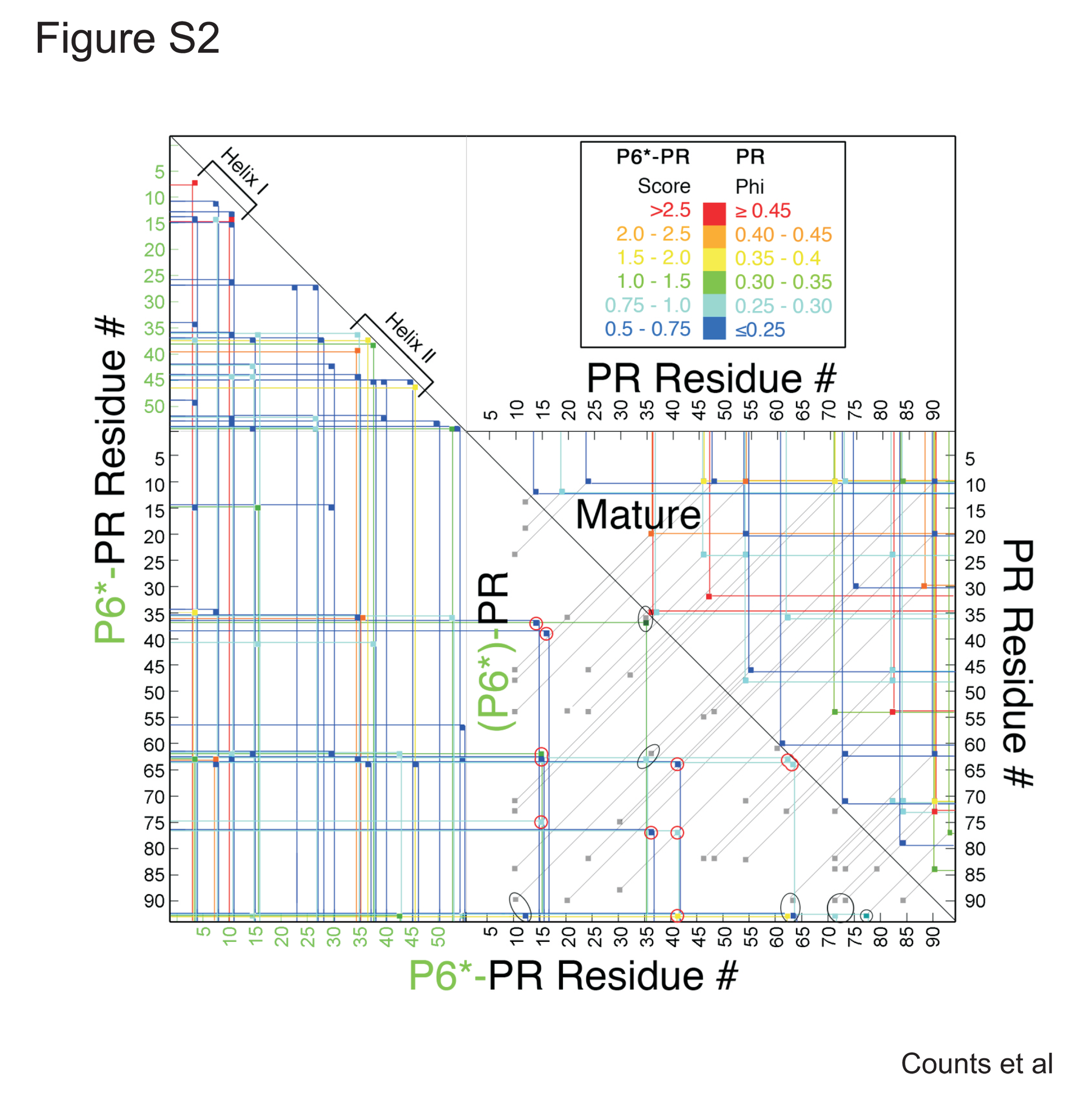

Supplement: S2 Fig — The covariance map for mature PR (lower right-hand corner) are mapped as residue-to-residue connections (residue # on both x- and y-axes, from 1–99, with the identical residue relationship along the main diagonal running from upper left-hand corner to lower right-hand corner). Lines trace the connections of covariance pairs (colored according to the Phi coefficients of Wu, et al [42]) and ending at the respective colored squares. The p6*-PR precursor covariance data are mapped on the left-hand side of the plot, with connecting lines and end point colored according to the scores defined in this study. The p6* domain and the mature PR are individually numbered as illustrated in Fig 1. Regularly spaced pairs near the main diagonal are indicative of helices, and are labeled as Helix I and II, respectively. The covariant pairs from the mature PR are transposed across the main diagonal into the PR region of the p6*-PR precursor (grey lines and squares). Pairs that are within 4 residues in sequence between the mature and precursor PR are indicated by the black circles, while pairs that are unique in this region to the precursor are indicated by red circles. (TIF) [file pone.0123561.s002.tif]

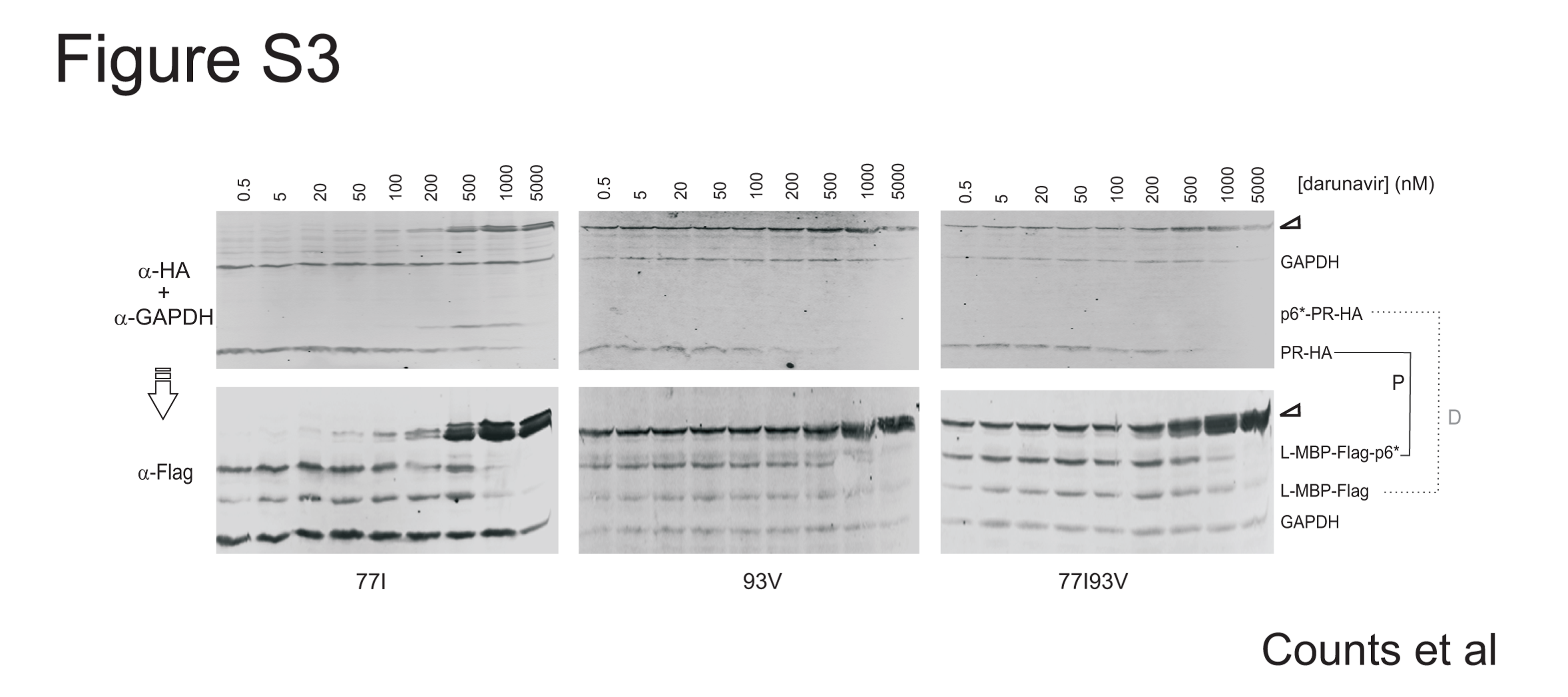

Supplement: S3 Fig — Transfected HEK293T cells were treated with the indicated concentrations of darunavir for ~15 h. The post-nuclear total lysates were then collected and resolved on SDS-PAGE. The resulting blot was first analyzed with HA and GAPDH antibodies followed by visualization by IR800 secondary antibody (upper image). The same blot was then re-probed with a Flag antibody to detect Flag-containing processing products (lower image). The full length precursor is indicated by triangles on the right. The proximal (P) and distal (D) cleavage products are connected by a solid and dashed line, respectively. (TIF) [file pone.0123561.s003.tif]
