# Supplementary material for: A Functional Interplay between Human Immunodeficiency Virus Type 1 Protease Residues 77 and 93 Involved in Differential Regulation of Precursor Autoprocessing and Mature Protease Activity
Source: PLoS One. 2015 Apr 20;10(4):e0123561. doi: 10.1371/journal.pone.0123561 (PMC4404164; doi:10.1371/journal.pone.0123561)
Supplement: S1 Table — a: covariance pairs are categorized into three interaction groups: p6*/p6*, p6*/PR, and PR/PR. The PR/PR covariance pairs with the involved amino acids far apart from each other per mature PR structure are highlighted in grey. (DOCX) [file pone.0123561.s004.docx]

Supporting Information Table S1.

Covariance pairs identified by OMES analysis of 147 drug naïve p6*-PR sequences^a^

| **Res of i** | **Res of j** | **S Score** | **Protein of i** | **Protein of j** |  | **Res of i** | **Res of j** | **S Score** | **Protein of i** | **Protein of j** |
| --- | --- | --- | --- | --- | --- | --- | --- | --- | --- | --- |
| N5 | P9 | 4.469 | p6* | p6* |  | N5 | E35 | 1.506 | p6* | PR |
| N5 | T39 | 0.841 | p6* | p6* |  | N5 | L63 | 1.005 | p6* | PR |
| N5 | L16 | 0.658 | p6* | p6* |  | N5 | I15 | 0.743 | p6* | PR |
| N5 | R36 | 0.545 | p6* | p6* |  | E12 | I62 | 0.752 | p6* | PR |
| N5 | A51 | 0.538 | p6* | p6* |  | E12 | L63 | 0.549 | p6* | PR |
| P9 | S17 | 0.924 | p6* | p6* |  | L16 | I93 | 0.959 | p6* | PR |
| P9 | E12 | 0.659 | p6* | p6* |  | L16 | I62 | 0.736 | p6* | PR |
| E12 | L16 | 3.793 | p6* | p6* |  | S17 | I15 | 1.251 | p6* | PR |
| E12 | G46 | 0.995 | p6* | p6* |  | S17 | R41 | 0.792 | p6* | PR |
| E12 | E15 | 0.703 | p6* | p6* |  | L31 | I62 | 0.616 | p6* | PR |
| E12 | F54 | 0.678 | p6* | p6* |  | L31 | I15 | 0.535 | p6* | PR |
| E12 | S38 | 0.641 | p6* | p6* |  | R36 | E35 | 2.003 | p6* | PR |
| E12 | S17 | 0.585 | p6* | p6* |  | R36 | M36 | 0.649 | p6* | PR |
| E12 | R28 | 0.563 | p6* | p6* |  | R36 | L63 | 0.642 | p6* | PR |
| E12 | N55 | 0.519 | p6* | p6* |  | S38 | I64 | 0.573 | p6* | PR |
| L16 | A44 | 0.972 | p6* | p6* |  | T39 | R41 | 0.866 | p6* | PR |
| L16 | G46 | 0.825 | p6* | p6* |  | A44 | I93 | 1.388 | p6* | PR |
| L16 | F56 | 0.731 | p6* | p6* |  | A44 | I62 | 0.908 | p6* | PR |
| L16 | T39 | 0.530 | p6* | p6* |  | G47 | I64 | 0.535 | p6* | PR |
| S17 | S38 | 0.824 | p6* | p6* |  | F54 | M36 | 0.777 | p6* | PR |
| N24 | R29 | 0.558 | p6* | p6* |  | F56 | I93 | 0.865 | p6* | PR |
| R28 | F56 | 0.943 | p6* | p6* |  | F56 | R57 | 0.559 | p6* | PR |
| R28 | F54 | 0.853 | p6* | p6* |  | F56 | L63 | 0.521 | p6* | PR |
| R28 | R29 | 0.726 | p6* | p6* |  | T12 | I93 | 0.644 | PR | PR |
| R29 | T39 | 0.508 | p6* | p6* |  | K14 | N37 | 0.546 | PR | PR |
| L31 | A44 | 0.501 | p6* | p6* |  | I15 | I62 | 1.095 | PR | PR |
| R36 | P41 | 2.411 | p6* | p6* |  | I15 | V77 | 0.856 | PR | PR |
| R36 | S38 | 0.897 | p6* | p6* |  | I15 | L63 | 0.604 | PR | PR |
| R36 | G46 | 0.541 | p6* | p6* |  | G16 | P39 | 0.664 | PR | PR |
| S38 | T39 | 1.766 | p6* | p6* |  | E35 | N37 | 1.020 | PR | PR |
| T39 | S40 | 1.185 | p6* | p6* |  | E35 | L63 | 0.794 | PR | PR |
| T39 | G47 | 0.668 | p6* | p6* |  | M36 | V77 | 0.735 | PR | PR |
| P41 | F54 | 0.600 | p6* | p6* |  | R41 | I93 | 1.824 | PR | PR |
| P41 | G47 | 0.509 | p6* | p6* |  | R41 | V77 | 0.868 | PR | PR |
| G46 | G47 | 0.500 | p6* | p6* |  | R41 | I64 | 0.643 | PR | PR |
| G47 | R48 | 1.645 | p6* | p6* |  | I62 | I93 | 1.680 | PR | PR |
| A51 | N55 | 0.616 | p6* | p6* |  | I62 | L63 | 0.856 | PR | PR |
| F54 | F56 | 1.035 | p6* | p6* |  | L63 | I64 | 0.955 | PR | PR |
| N55 | F56 | 0.544 | p6* | p6* |  | L63 | I93 | 0.697 | PR | PR |
|  |  |  |  |  |  | V71 | I93 | 0.876 | PR | PR |
|  |  |  |  |  |  | **V77** | **I93** | **0.984** | **PR** | **PR** |

^a^: covariance pairs are categorized into three interaction groups: p6*/p6*, p6*/PR, and PR/PR. The PR/PR covariance pairs with the involved amino acids far apart from each other per mature PR structure are highlighted in grey.
